# Construction of a Glutathione-Responsive and Silica-Based Nanocomposite for Controlled Release of Chelator Dimercaptosuccinic Acid

**DOI:** 10.3390/ijms19123790

**Published:** 2018-11-28

**Authors:** Hongqiang Zhai, Yuli Wang, Menghua Wang, Shuai Liu, Feifei Yu, Chunsheng Gao, Guiling Li, Qiang Wu

**Affiliations:** 1 College of Pharmacy, Institutes of Environment and Medicine, Henan University, Kaifeng 475004, China; zhaihongqiang92@163.com (H.Z.); 104753160940@vip.henu.edu.cn (M.W.); 104754170887@vip.henu.edu.cn (S.L.); 2 Institute of Medicinal Biotechnology of Medical Science & Peking Union Medical College, Beijing 100850, China; Yufeifei760@126.com; 3 State key laboratory of toxicology and medical countermeasures, Beijing Institute of Pharmacology and Toxicology, Beijing 100850, China; Wangyuli764@126.com (Y.W.); gaocs@bmi.ac.cn (C.G.)

**Keywords:** mesoporous silica, disulfide bond, glutathione-responsive, controlled release, dimercaptosuccinic acid

## Abstract

Dimercaptosuccinic acid (DMSA) is an oral heavy metal chelator. Although DMSA is the most acceptable chelator in the urinary excretion of toxic elements from children and adults, its defects in plasma binding and the membrane permeability limit its interaction with intracellular elements and affect its efficacy in chelation therapy. Herein, a novel nanocomposite composed of mesoporous silica nanoparticles (MSNs), disulfide bond, and DMSA was synthesized and characterized with a scanning/transmission electron microscope, IR and Raman spectra, and TGA analysis. The in vitro interactions with glutathione (GSH) and cellular uptake assays showed that it was able to be stable in extracellular environments such as in blood, be internalized by cells, and release DMSA inside via GSH-triggered disulfide cleavage reaction. The in vitro adsorption assays showed that MSNs-SH as its intracellular metabolite had strong adsorbability for models of Hg^2+^ or Pb^2+^. The hemolysis and cell viability assays showed that it was compatible with blood and cells even at a concentration of 1000 μg·mL^−1^. All above could not only enable it to be a GSH-responsive drug delivery system (DDS) for DMSA delivery but also to be a solution for its defects and efficacy. Thus, introduction of intelligent DDS might open a new avenue for DMSA-based chelation therapy.

## 1. Introduction

Multiple industrial, domestic, agricultural, medical, and technological applications have caused wide distribution of heavy metal elements in the environment such as lead (Pb), mercury (Hg), and arsenic (As) [1]. These elements can enter into the human body through the food chain, water, soil, and air and accumulate over a long time in tissues and organs [1,2]. These elements have no essential biochemical roles but can induce multiple organ damage as they bind with cell components such as the cell membrane, mitochondrial, lysosome, endoplasmic reticulum, nuclei, and enzymes [3]. Current research further implicates them as a contributing etiologic factor in a number of diseases such as cardiovascular and renal disease, neurological decline, and tumors [1,2,3,4].

Chelation therapy is a well-documented method used for heavy metal poisoning. Among common chelators, dimercaptosuccinic acid (DMSA) is the most acceptable one in the urinary excretion of above toxic elements from children and adults [1]. DMSA is generally administrated by the oral route. Oral absorption is reported to be approximately 20% with most DMSA in plasma being protein bound (95%, mainly to albumin). Only a very small amount is present as free drugs [5,6,7]. DMSA possesses defects of high plasma binding and low membrane permeability, which cause its approach to the targeted tissues and subsequent chelation with intracellular elements limited. To show activity, an excess amount and frequent administration are often needed for clinic applications of this chelator [1], which not only may burden and damage the renal system as metal chelates are cleared from the body but also produce a number of detrimental effects accrued from extraction of essential metals [8,9]. The information above is also why DMSA is used for treatment of acute poisoning and not for non-acute sub-poisoning (chronic accumulation of toxic elements in tissues and organs). Thus, the method that can overcome DMSA defects and increase its efficacy is highly desirable in chelation therapy. Unfortunately, seldom efforts are made in this regard beside structural modification with the monoisoamyl group or amino acids [10,11].

The use of nanocarriers for drug delivery has received a great deal of attention in the past several decades [12,13]. Nanocarriers can be distributed systemically via intravenous injection, which is internalized by cells and locally delivered into a particular tissue. Nanocarriers with stimuli-responsive groups (such as group sensitive to pH, redox potential, temperature, photo-irradiation, and biomacromolecule) can achieve near zero-premature drug release in blood circulation and smart drug release in the targeted tissues [14,15,16,17,18,19,20]. This technique of controlled release is believed to be highly efficient in mitigating systemic toxicity and enhancing therapeutic efficacy of therapeutic agents and has been proposed for many therapeutic applications including treatment of cancer and central neural system disorders as well as cardiac tissue repair [21]. Yet, its attempt in heavy metal poisoning is still inferior.

**Scheme 1:**
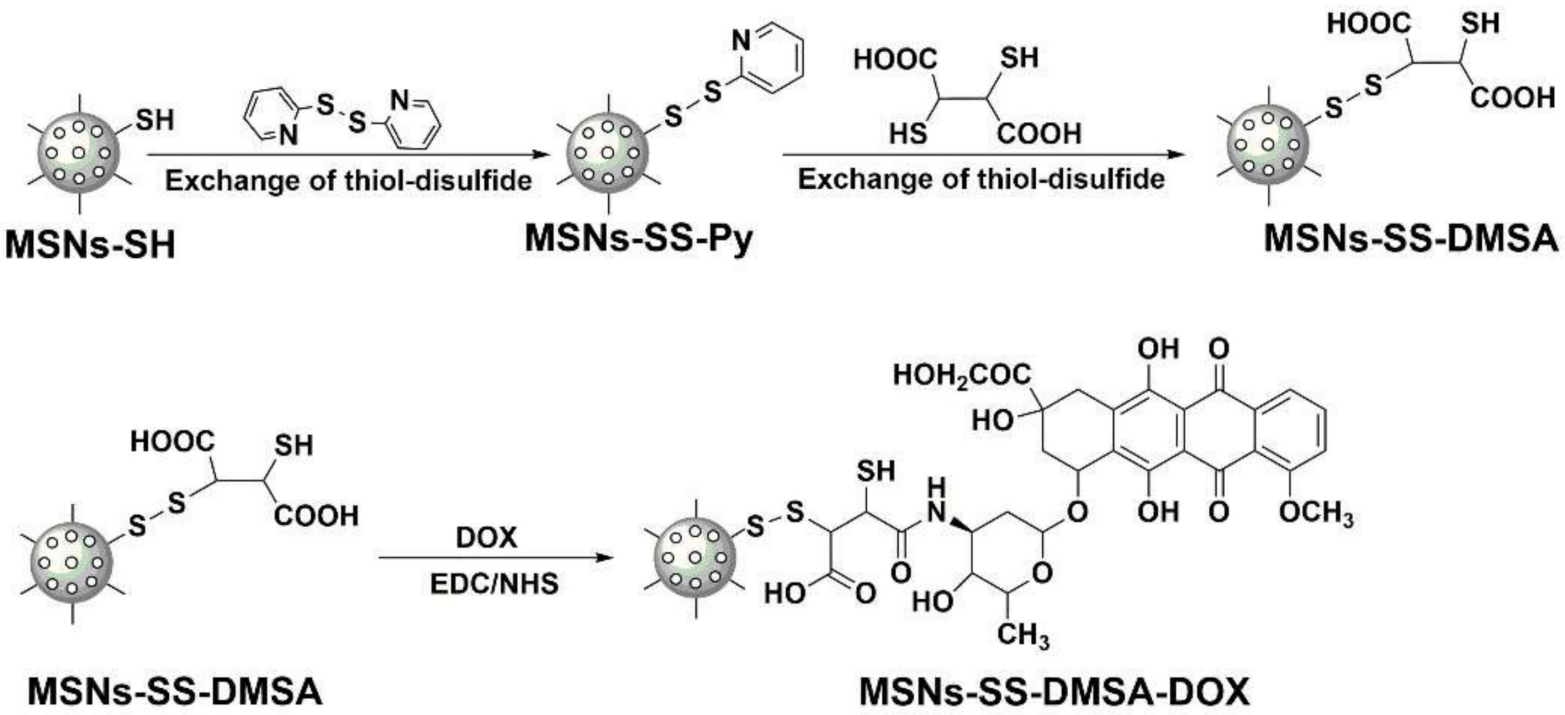
Synthesis of DMSA-modified or DOX-labelled MSNs derivatives.

**Scheme 2:**
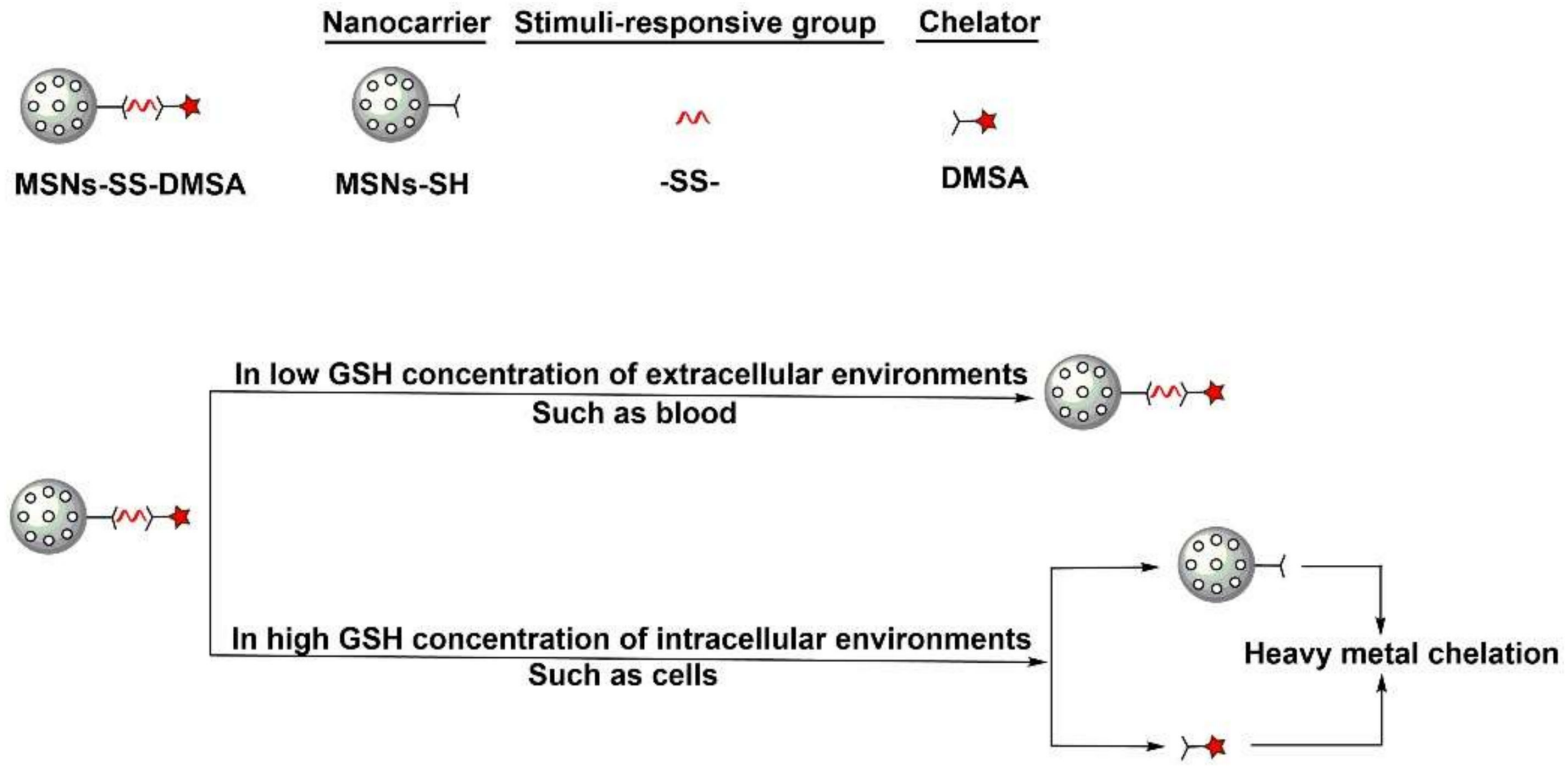
The intact of MSNs-SS-DMSA in extracellular environments and GSH−responsive drug release in intracellular environments.

In this study, we covalently conjugated DMSA on the surfaces of mesoporous silica nanoparticles (MSNs) via disulfide bonds. The thiol-modified MSNs were denoted as MSNs-SH. The DMSA—modified MSNs-SH, which was obtained by thiol-disulfide exchange reactions, was denoted as MSNs-SS-DMSA (Scheme 1). The aim was to construct a smart nanocomposite for DMSA delivery and offer a solution for its defects and efficacy. In this nanocomposite, we chose MSNs as the nanocarrier component and disulfide bond as the stimuli-responsive component in view of previous studies [22,23]. MSNs has been extensively investigated for drug delivery applications due to unique advantages such as large surface area, high pore volume, easy chemical functionalization, and good biocompatibility. Recent advances have demonstrated that MSNs-based controlled release systems have the potential for effective stimuli-responsive drug delivery to maximize desired therapeutic responses [22]. The disulfide bond is an oxidizing group and sensitive to in vivo glutathione (GSH). Generally, the disulfide bond is relatively stable in a low GSH concentration of extracellular environments (0.02–0.04 mM) such as in blood and readily cleaved in high GSH concentration of intracellular environments (1–10 mM), which enable it to be an appealing linker for the design of an intelligent drug delivery system (DDS) [24,25,26]. Conjugation of DMSA with these two components was to construct a GSH-responsive nanocomposite, which involves constructing a nanocomposite that was able to be stable in extracellular environments, be internalized by cells, and release DMSA inside via a GSH-triggered disulfide cleavage reaction (Scheme 2). If successful, loss of DMSA due to plasma binding on transport could be avoided by GSH-responsive controlled release and defect of this chelator in transmembrane could be improved by nanocarrier-mediated penetration. In addition, after the intracellular release of DMSA, the remaining product of the as-synthesized nanocomposite would be MSNs-SH (Scheme 2). MSNs-SH was a sulfhydryl-modified MSNs. This characteristic MSNs have been reported to be highly efficient in removing heavy metals from different environment systems such as the aquatic environment and biological fluids (blood, urine, synthetic gastric fluid (SGF), and synthetic intestinal fluid (SIF)) [9,27,28,29,30,31]. Thus, presence of MSNs-SH in cells might be helpful for DMSA-based heavy metal detoxification. In addition, other information of this nanocomposite might also be helpful of its application. Take excretion of heavy metals as an example. It is well-known that DMSA and MSNs-SH can be excreted from urine and feces [9,24] and, thus, it is possible that the accumulated heavy metals can also be excreted in these ways after their complexation with these components.

## 2. Results and Discussion

**Figure 1:**
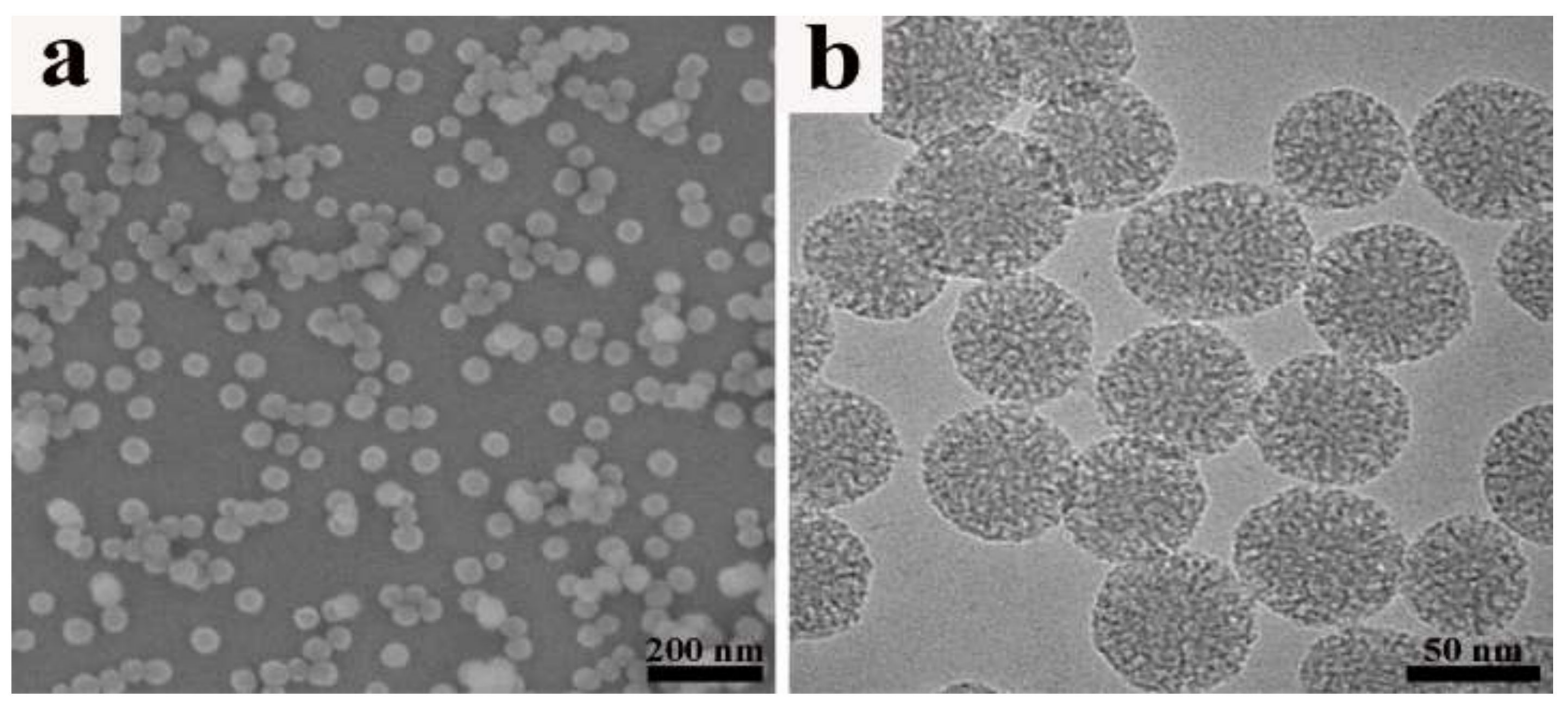
SEM (**a**) and TEM (**b**) images of MSNs-SH.

**Figure 2:**
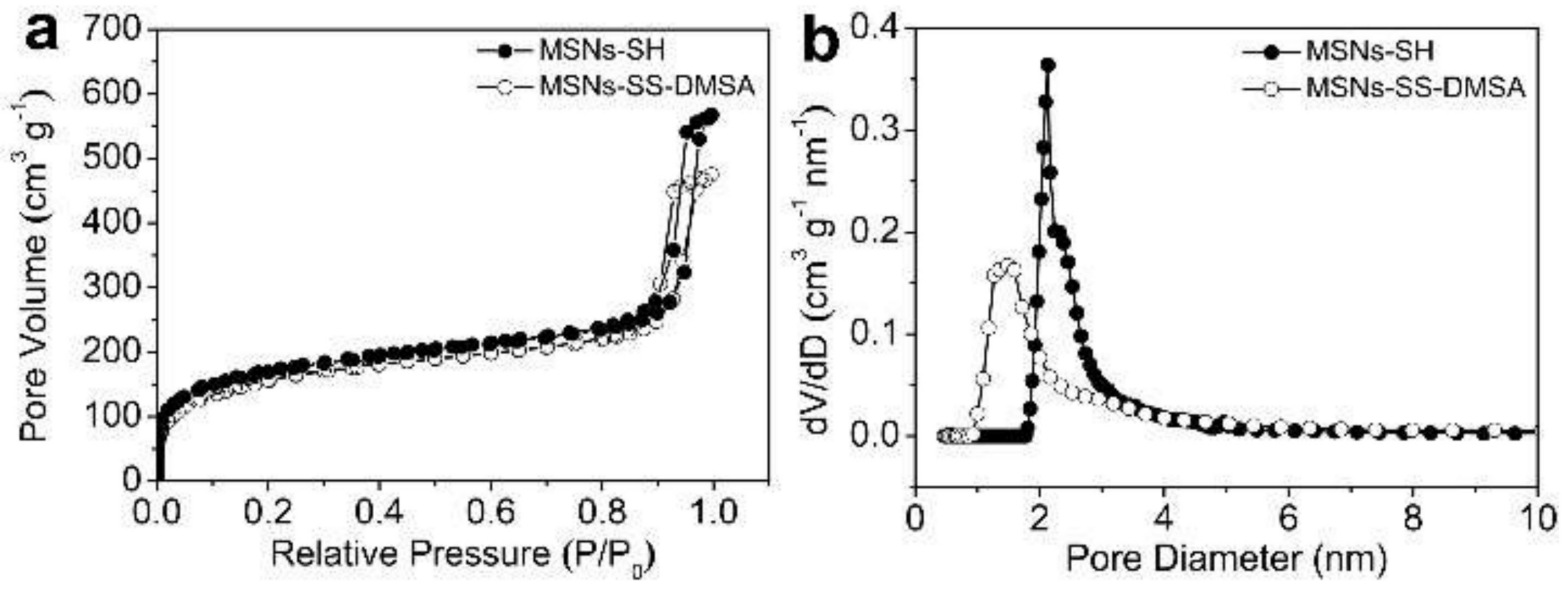
N_2_ adsorption/desorption isotherms (**a**) and pore size (**b**) distributions of MSNs derivatives.

**Figure 3:**
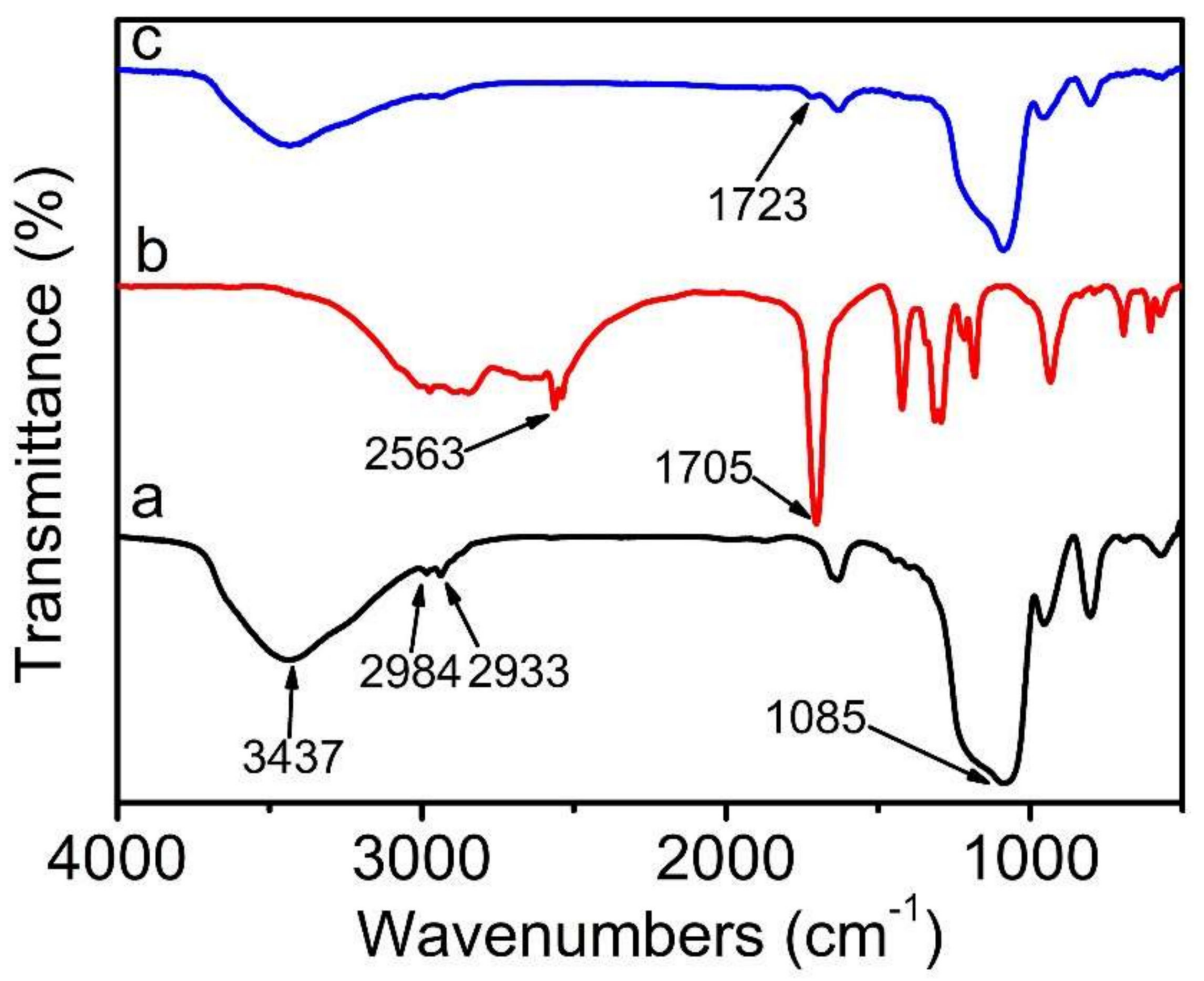
FTIR spectra of MSNs-SH (**a**), DMSA (**b**), and MSNs-SS-DMSA (**c**).

**Figure 4:**
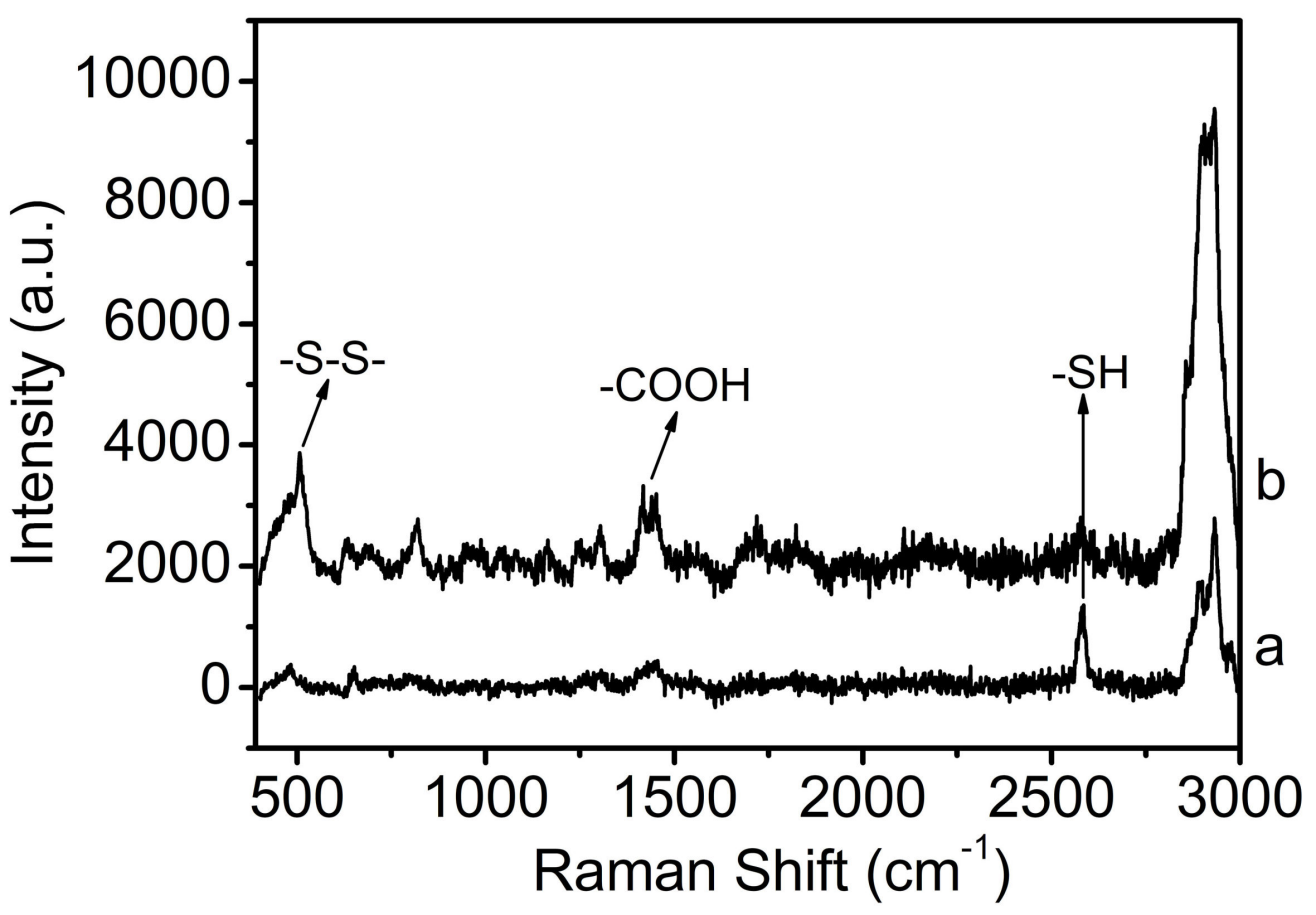
Raman spectra of MSNs-SH (**a**) and MSNs-SS-DMSA (**b**).

**Figure 5:**
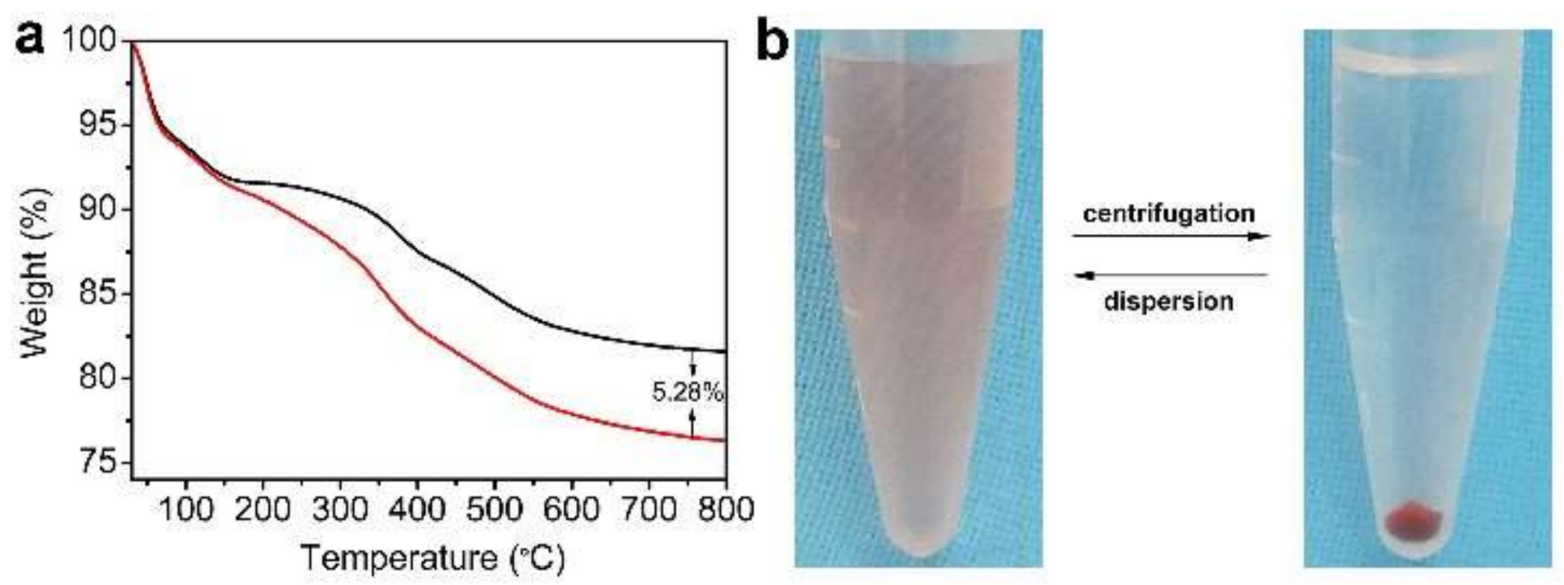
(**a**) TGA curves of MSNs-SH (black) and MSNs-SS-DMSA (red); (**b**) the dispersity of DOX−labeled MSNs-SS-DMSA in PBS buffer solution (pH 7.4).

MSNs-SH particles were synthesized by a modified one-pot method, according to previous reports, in which TEOS was used as a silica source, CTAB and Brij-58 as the structure-directing agents, and MPTES as a co-condensation agent [32,33,34]. Conjugation of DMSA with MSNs-SH was carried out via thiol-disulfide exchange reactions and mediated by 2, 2-dipyridyl disulfide (Py-SS-Py, Scheme 1). The intermediate product was denoted as MSNs-SS-Py. Py-SS-Py was a common agent used for thiol-disulfide exchange reactions. The reaction could be monitored by color change of the solution. Generally, yellow Py-SH solution would be yielded after a reaction. As shown in SEM and TEM images (Figure 1a,b), MSNs-SH alone displayed a very uniform size distribution around 50 nm, a regular spherical morphology, a disordered porous structure, and a perfect mono-dispersity. Morphology of MSNs-SH had no significant change after its reaction with DMSA, but its BET surface area, pore volume, and pore size were reduced from 621 cm^2^·g^−1^, 0.88 cm^3^·g^−1^, and 2.18 nm to 565 cm^2^·g^−1^, 0.73 cm^3^·g^−1^, and 1.48 nm respectively (Figure 2a,b). The conjugation procedure was further characterized by FTIR and Raman spectra. It could be seen from Figure 3 and Figure 4 that MSNs-SH alone showed clear FTIR signals at the wavelength of 2984 and 2933 (−CH_2_) and 1085 cm^−1^ (Si−O−Si) and a clear Raman signal at the wavelength of 2600 cm^−1^ (−SH) [35]. After thiol−disulfide exchange reactions, a new FTIR signal at the wavelength of 1723 cm^−1^ (−COOH) and two new Raman signals at the wavelength of 500 cm^−1^ (−SS−) and 1400 cm^−1^ (−COOH) were observed [35]. Moreover, appearance of the −SS− signal was accompanied with marked attenuation of −SH signal. The data suggested successful conjugation of DMSA with MSNs via the disulfide bond and its efficiency was 5.28% (*w*/*w*), which is roughly estimated by TGA analyses (Figure 5a). The obtained nanocomposite has been denoted as MSNs-SS-DMSA above. No −SH signal (2563 cm^−1^) that could be seen clearly from FTIR spectra of DMSA (Figure 3b) was found from that of MSNs-SH and no −SS− signal (1420 cm^−1^) was found from that of MSNs-SS-DMSA, which might be due to the weak signal intensity. Furthermore, zeta (ζ) potentials and particle sizes of MSNs derivatives were measured in PBS buffer solution (pH 7.4) using dynamic light-scattering method. The data showed that MSNs-SH had a negative ζ potential of −24.2 mV, which was due to the existence of dehydrogenized silanol groups on its surface and had a larger particle size (91.2 nm) than that observed from SEM and TEM, which was due to slight particle aggregation and/or hydration of the particle surface [25]. After DMSA conjugation, ζ potential and particle size were increased to −42.7 mV and 125.0 nm, respectively, due to the contribution of negatively charged and hydrophilic carboxyl-groups, which suggests that MSNs-SS-DMSA possessed good colloidal stability under the physiological environment. The good stability of this MSNs derivative was further verified in cell culture media in which no particle aggregation event occurred.

**Figure 6:**
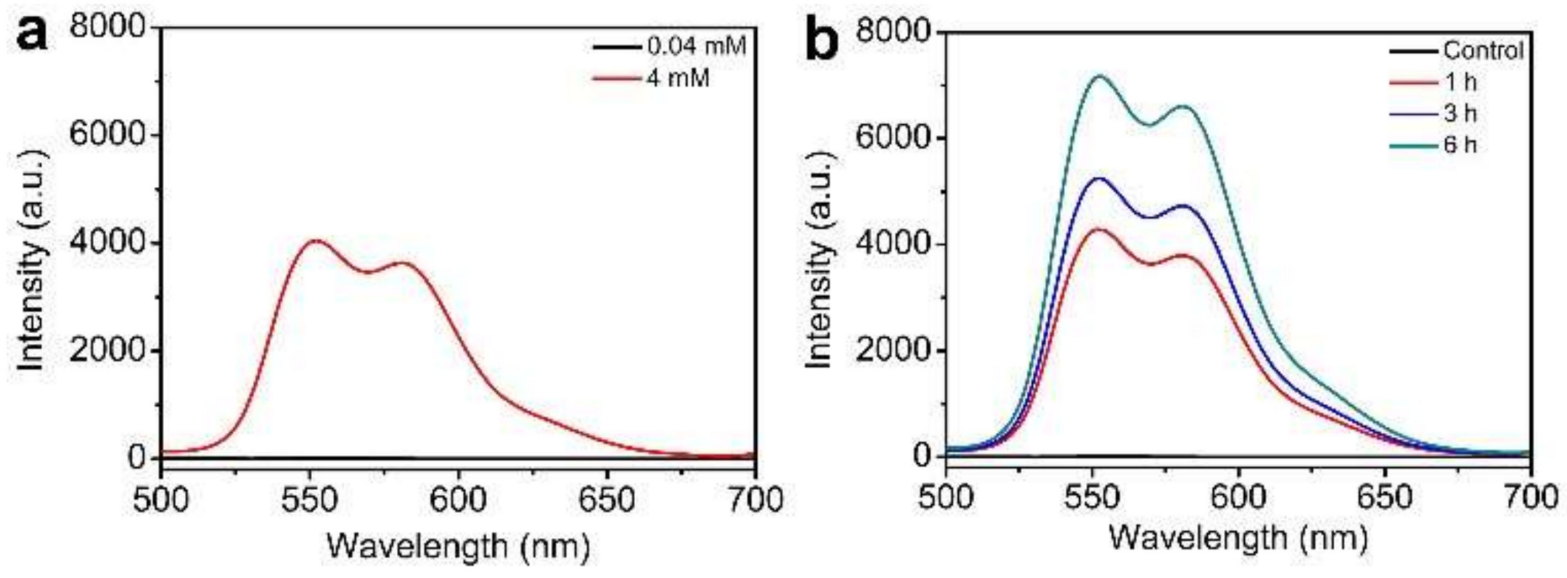
Fluorescence spectra of the supernatants resulted from interactions between DOX-labeled MSNs-SS-DMSA and varied concentrations of GSH for 1 h (**a**) and from interactions between the above labeling product and 4 mM of GSH (**b**) for different times.

**Figure 7:**
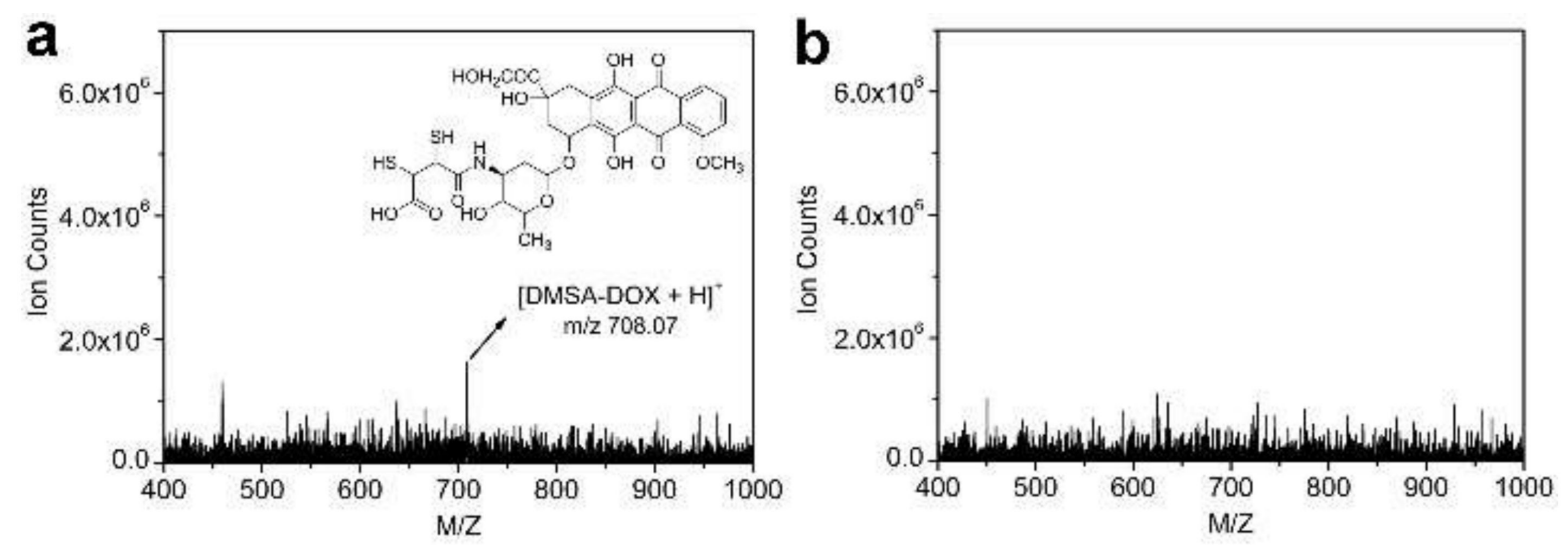
Mass spectra of the supernatants extracted from 1 h interactions between DOX-labeled MSNs-SS-DMSA and 4 mM and (**a**) 0.04 mM (**b**) of GSH.

To verify GSH-responsiveness of MSNs-SS-DMSA, doxorubicin (DOX) was selected as a fluorescent tag for labeling this nanocomposite. First, there is only one NH_2_ group present in doxorubicin and, second, the DOX-DMSA conjugate was obtained via amide formation using the carbodiimide coupling agent (Scheme 1). The obtained product was denoted as MSNs-SS-DMSA-DOX. If such labeling were achieved, release of DMSA would be monitored by release of DMSA-DOX from MSNs-SS-DMSA-DOX. After amide formation, red particles with good dispersity in PBS buffer solution (Figure 5b) were obtained by exhausted dialysis against methanol solution until no DOX fluorescence was detected from the supernatant, which was to efficiently remove those physically adsorbed DOX from MSNs-SS-DMSA. In this case, it should be noted that EDC/NHS agents used in a carbodiimide reaction had no effect on disulfide activity theoretically and disulfide bonds in the framework of MSNs-SS-DMSA should be intact after DOX labeling. Subsequently, these red particles were incubated with varied concentrations of GSH in PBS buffer solution and then the supernatants were collected for fluorescence detection. As shown in Figure 6a, when 0.04 mM of GSH (represented as a high extracellular concentration) was used, no fluorescence signal was detected from the supernatant, but when 4 mM of GSH (represented as a low intracellular concentration) was used, fluorescence signals at the wavelengths of 550 and 580 nm were clearly detected and their intensities enhanced gradually with extension of incubation time (Figure 6b), which suggests that GSH even at a low intracellular concentration was able to interact with those red particles and leads to a time-dependent release of a fluorescence product, but GSH even at a high extracellular concentration was not. To understand structural information of the above released product, the supernatants was presented for LC−MS analyses. As shown in Figure 7, a strong signal (*m*/*z* 708.07) corresponding to protonated DMSA-DOX (C_31_H_33_O_14_NS_2_) was found in mass spectra of supernatant containing 4 mM of GSH (Figure 7a) and no corresponding signal was found in that of the supernatant containing 0.04 mM of GSH (Figure 7b). In this case, it should be noted that DOX also exhibited fluorescence emission at the above wavelengths and a possibility that the released product originated from those physically adsorbed DOX should not be ruled out. Fortunately, no signal (*m*/*z* 543.53) corresponding to protonated DOX (C_27_H_29_O_11_N) was found in the above mass spectra plots, which suggests that the released product was DMSA-DOX rather than DOX. Since DMSA-DOX only originated from disulfide-cleavage of MSNs-SS-DMSA-DOX, its verification in the supernatant containing 4 mM of GSH suggested that the desirable DOX labeling of MSNs-SS-DMSA was achieved. Furthermore, the results of in vitro interactions between MSNs-SS-DMSA-DOX (called red particles above) and different concentrations of GSH indicated that MSNs-SS-DMSA was sensitive to GSH and was able to be intact in low GSH concentration of extracellular environments and release DMSA in a high GSH concentration of intracellular environments.

**Figure 8:**
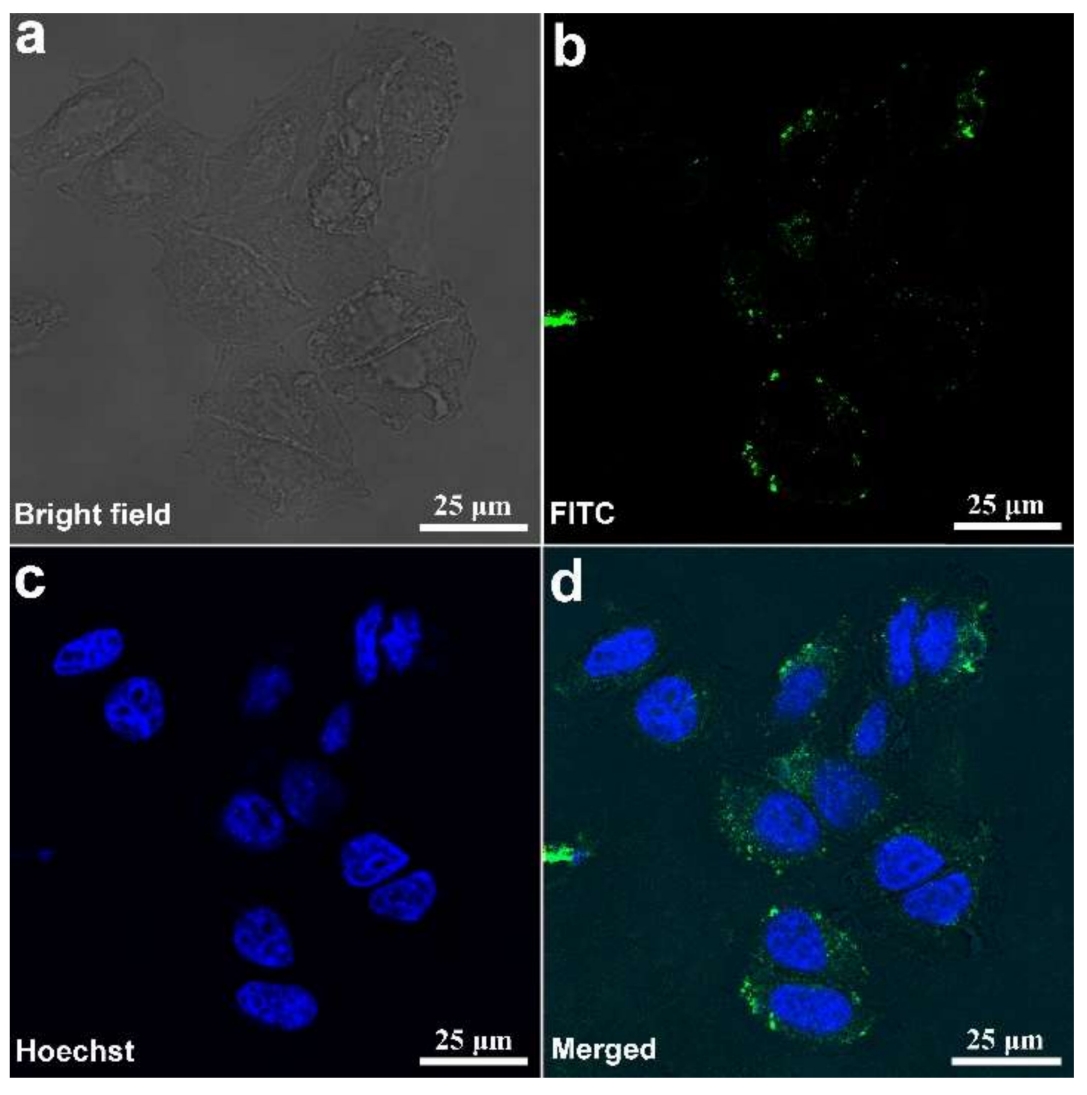
LSCM images of HL-7702 cells after incubation with FITC-labeled MSNs-SS-DMSA for 6 h.

**Figure 9:**
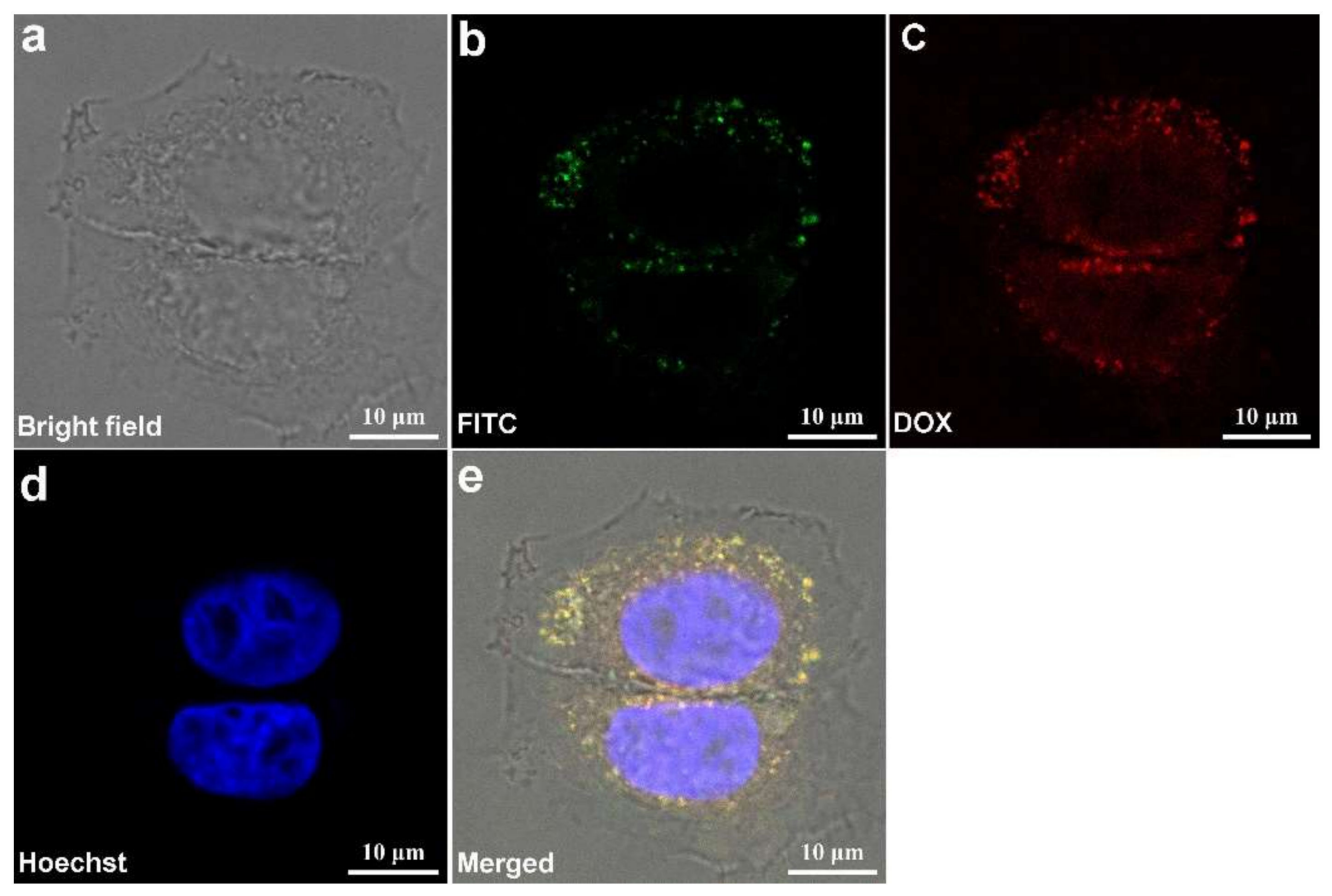
LSCM images of HL-7702 cells after incubation with FITC-labeled MSNs-SS-DMSA-DOX for 6 h.

The good in vitro GSH-responsiveness of MSNs-SS-DMSA intrigued us to further investigate its intracellular responsiveness. To this end, this nanocomposite was labeled with FITC or FITC/DOX, according to schedules described in the following experimental section. The obtained products were denoted as FITC-labeled MSNs-SS-DMSA and MSNs-SS-DMSA-DOX, respectively. The zeta potentials of these two labeling products were 43.5 mV and 40.8 mV, respectively, which suggests no significant changes of these particles in colloidal dispersity after FITC incorporation. The preparation of these two labeling products was initiated from that of FITC-labeled MSNs-SH (co-condensation of FITC-labeled APTES and MPTES during sol-gel reaction of MSNs) and then the next modifications for this labeling product were similar to the above label-free products. In addition, it should be noted that, as liver tissue was the largest repository (33%) of heavy metals among the soft tissues when heavy metals entered into the human body, a normal human liver cell line HL-7702 was selected for following the investigations. Figure 8 showed LSCM images of HL-7702 cells after their incubation with FITC-labeled MSNs-SS-DMSA for 6 h at 37 °C. It could be seen that green fluorescence of FITC was observed in the cytoplasm and blue fluorescence of Hoechst 33342 was observed in the nucleus (Figure 8b,c), which suggests that FITC-labeled MSNs-SS-DMSA was able to penetrate into cells and its distribution was the main in cytoplasm and not in the nucleus. Figure 9 showed LSCM images of HL-7702 cells after their incubation with FITC-labeled MSNs-SS-DMSA-DOX in the same condition as that of FITC-labeled MSNs-SS-DMSA. It could be seen that green fluorescence of FITC was still observed in cytoplasm (Figure 9b), but red fluorescence of DOX was observed not only in the cytoplasm but also in the nucleus (Figure 9c). These fluorescence distributions were further confirmed by the merged images in which yellow fluorescence due to the overlapping of red fluorescence spots with green fluorescence spots was observed in the cytoplasm and light-purple fluorescence due to the overlapping of red fluorescence spots with blue fluorescence spots was observed in the nucleus (Figure 9e). The absence of green fluorescence in the nucleus suggested that FITC-labeled MSNs-SS-DMSA-DOX also could not penetrate the nucleus and red fluorescence in the nucleus did not originate from particle emission. With respect to the origin of such fluorescence, only following analysis could present a reasonable explanation. After cellular uptake of FITC-labeled MSNs-SS-DMSA-DOX, its disulfide bonds were cleaved by intracellular GSH. The released DMSA-DOX being a free DOX derivative could penetrate the nucleus and enable it to show red fluorescence. DOX was a DNA intercalator and its action site was mainly in the nucleus. Therefore, a conclusion could be made that MSNs-SS-DMSA as a parent of the above FITC-labeled products could also penetrate the cells and release DMSA inside in a GSH-responsive way.

**Figure 10:**
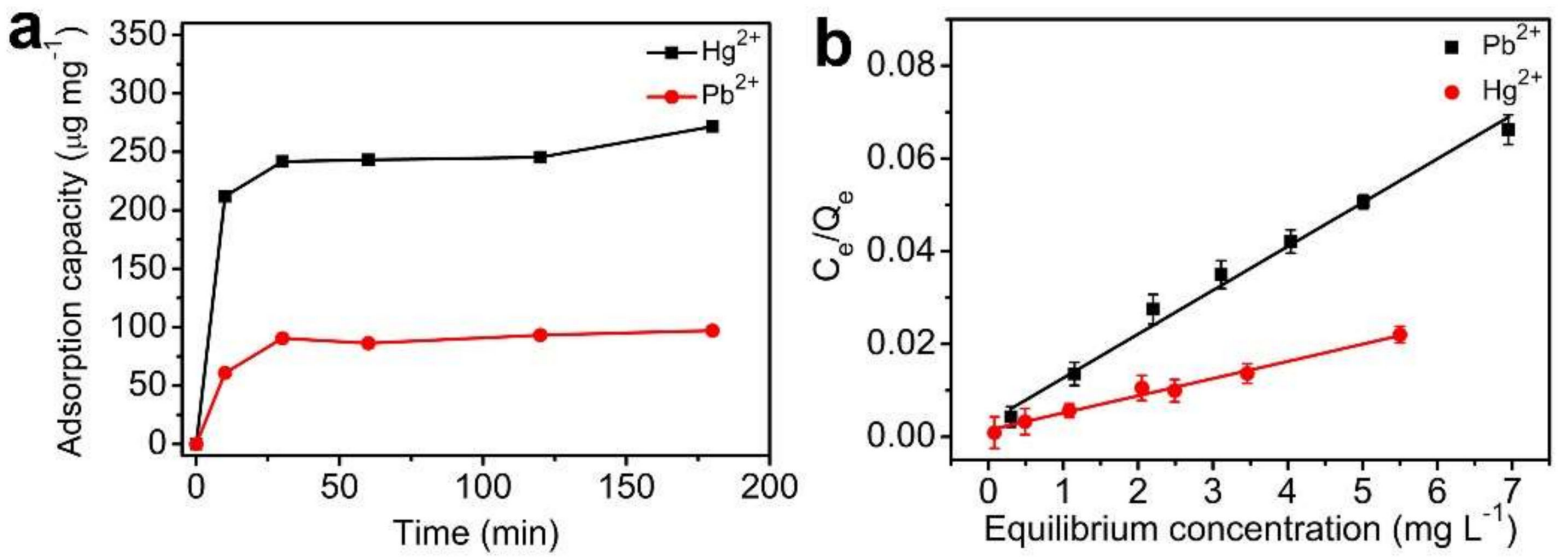
Adsorption kinetic and isotherm of heavy metals on MSNs-SH.

**Figure 11:**
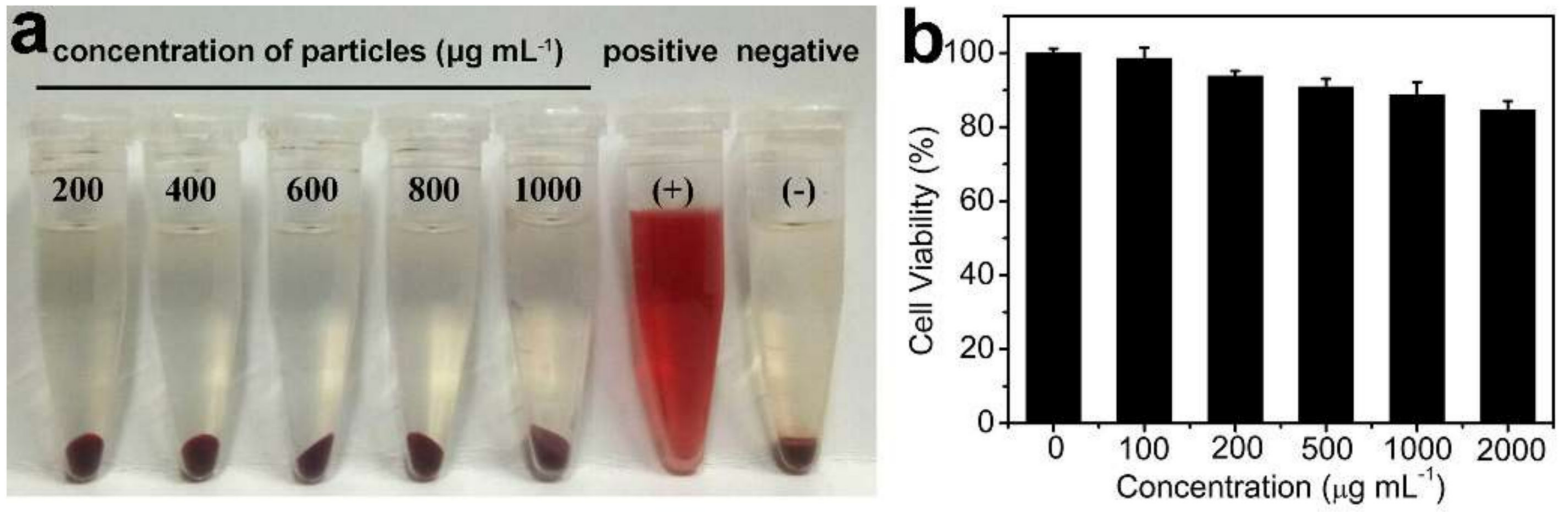
Photographs of RBCs after incubation with varied concentrations of MSNs-S-S-DMSA for 24 h (**a**). RBCs samples treated with deionized water and DPBS were used as the positive and the negative control, respectively. Viability of HL-7702 cells after incubation with varied concentrations of MSNs-SS-DMSA for 24 h (**b**).

After demonstration of intracellular DMSA release, in vitro adsorption assays were carried out to mimic interactions between MSNs-SH (intracellular metabolite of MSNs-SS-DMSA) and heavy metals. Figure 10a showed adsorption kinetic of heavy metal Hg^2+^ and Pb^2+^ on MSNs-SH. It could be seen that MSNs-SH showed rapid adsorption for these two metals in the first 20 min and then its adsorption process decreased gradually until the equilibrium was achieved. The saturated adsorption was approximate 263.3 μg mg^−1^ for Hg^2+^ and 108.2 μg·mg^−1^ for Pb^2+^, according to the study of adsorption isotherm (Figure 10b). For more details, please see the experimental section. The results suggested that MSNs-SH had strong adsorbability for heavy metals and such ability might enable it to play a synergic role in heavy metal chelation with the released DMSA despite the limitation of intracellular environments for particle mobility. Lastly, hemolysis and cell viability assays were performed to investigate biocompatibility and cytotoxicity of MSNs-SS-DMSA. As shown in Figure 10b and Figure 11, no hemolysis events occurred when incubating blood with even 1000 μg·mL^−1^ of particles for 24 h and over 85% cell viability (MTT assays) was achieved after incubating HL-7702 cells with even 1000 μg·mL^−1^ of particles for 24 h. The results were in agreement with those of DMSA conjugated Fe_3_O_4_@SiO_2_ [36], which suggests that MSNs-SS-DMSA possessed good biocompatibility.

## 3. Materials and Methods

### 3.1. Materials

Cetyltrimethyl ammonium bromide (CTAB), tetraethoxysilane (TEOS), 2, 2-dipyridyl disulfide (Py-SS-Py, 98%), Brij-58, DMSA (98%), and Doxorubicin (DOX) were obtained from Aladdin Co., Ltd. (Shanghai, China). 1-ethyl-3-(3-dimethylaminopropyl) carbodiimide hydrochloride (EDC), *N*-hydroxy-succinimide (NHS) and fluorescein isothiocyanate (FITC) were obtained from Sigma-Aldrich. 3-mercaptopropyltriethoxy-silane (MPTES) and 3-aminopropyltriethoxysilane (APTES) were obtained from Beijing J&K Co., Ltd. Fetal calf serum (FBS) and Dulbecco′s modified eagle medium (DMEM) were purchased from GIBCO, Invitrogen Co., Ltd. (Carlsbad, CA, USA). All other chemicals are of an analytical grade.

The HL-7702 cell line was obtained from Shanghai Cell Bank, Chinese Academy of Sciences (CAS), and was cultured in DMEM supplemented with 10 % FBS at 37 °C in a humidified atmosphere with 5% CO_2_.

### 3.2. Synthesis of MSNs-SH

MSNs-SH was synthesized, according to previous reports [32,33,34]. CTAB (0.45 g) and Brij58 (0.47 g) were dissolved in 120 mL of phosphate buffer solution (PBS, pH 7.0). Afterward, TEOS (2.14 mL) and MPTES (180 μL) were added slowly. The obtained suspension was stirred at 60 °C for 8 h. Lastly, the obtained products were separated by centrifugation and washed by ethanol at least three times. To remove the CTAB template, the above products were refluxed in a mixed solution of ethanol (150 mL) and hydrochloric acid (2 mL) for 24 h. After that, the surfactant was removed out by centrifugation and the obtained solid power were washed and dried under vacuum overnight.

### 3.3. Synthesis of MSNs-SS-DMSA and Its Labeling Products

The synthesis of MSNs-SS-DMSA was performed by two steps of thiol-disulfide exchange reactions. Py-SS-Py (600 mg) was added into methanol solution (25 mL) of MSNs-SH (100 mg). The suspension was stirred for 24 h at room temperature in the dark. After the reaction, the obtained solid power were separated by centrifugation and washed by methanol at least six times. Afterward, DMSA (100 mg) was added into the methanol solution (25 mL) of the above solid power. The suspension was stirred for 24 h at room temperature in the dark. Lastly, the obtained products were collected by centrifugation and washed as described above.

DOX-labeled MSNs-SS-DMSA was prepared by carbodiimide reaction. EDC (287.4 mg) and NHS (172.8 mg) were added into the PBS buffer solution (20 mL, 0.1 M, and pH 5.3) of MSNs-SS-DMSA power (50 mg). After 2 h of magnetic stirring, the pH value of the above suspension was adjusted to 8.3 with NaOH (0.5M) and 5 mg of DOX was added in. After that, the suspension was continually stirred for 4 h at room temperature in the dark. Dialysis against the methanol solution was necessary for the final solid product in which no fluorescence of DOX was detected from the supernatant. The aim was to remove those physically adsorbed DOX.

FITC-labeled MSNs-SS-DMSA and FITC-labeled DOX derivative of MSNs-SS-DMSA were prepared according to the following schedules. First, 8.6 mg of FITC was mixed with 0.197 mL of APTES in anhydrous ethanol (2 mL) for 24 h at room temperature [33]. Afterward, the obtained FITC-labeled APTES (50 μL) was added with MPTES (180 μL) at the co-condensation stage of sol−gel reaction of MSNs as described above. Lastly, the obtained FITC-labeled MSNs-SH was continually conjugated with DMSA and DOX as described above.

### 3.4. In vitro Interactions of DOX-Labeled MSNs-SS-DMSA with GSH

A total of 10 mg of DOX-labeled MSNs-SS-DMSA was added into 2 mL of PBS buffer solution (pH 7.4) containing different concentrations of GSH. The obtained suspensions were shaken in a water bath at 37 °C. At certain time intervals, the particles were centrifugally separated and the supernatants were collected for fluorescence detection and LC−MS analysis. In this study, 0, 0.04, and 4 mM were selected as the concentrations of GSH. The selection of 0.04 mM and 4 mM was to stimulate low GSH concentrations of extracellular environments and high GSH concentrations of intracellular environments, respectively.

### 3.5. Cellular Uptake of FITC-Labeled MSNs Derivatives

HL-7702 cells were seeded onto a round glass cover slips in 24-well tissue culture plate at a density of 5 × 10^4^ cells per well. After 12 h of attachment, HL-7702 cells were treated with 2 μg·mL^−1^ of FITC-labeled MSNs derivatives. After 6 h, the nuclei was stained by Hoechst 33258 for 15 min. Observation of the fixed cells was conducted using the laser scanning confocal microscope (LSCM) (FV1000, Olympus, Tokyo, Japan). The excitation wavelengths of FITC and DOX were 490 nm and 470 nm, respectively.

### 3.6. Adsorption of Heavy Metals on MSN-SH

A volume of 100 mL PBS buffer solutions (pH 7.4) containing 5 mg·L^−1^ of Hg^2+^ and Pb^2+^ for each were added with 1 mg of MSN-SH and agitated in a rotator oscillator at 37 °C for 3 h to study the adsorption kinetics of the adsorbent. The sample solutions were collected at certain intervals of 10, 30, 60, 120, and 180 min. After centrifugation separation of the particles, the supernatants were spiked with HNO_3_ to 3% (*v*/*v*) and then injected to ICP (iCAP 7200 Duo, Thermo Scientific, Waltham, MA, USA) to determine the final concentration of Hg^2+^ and Pb^2+^.

To derive data for the adsorption isotherm, a series of 100 mL PBS buffer solutions (pH 7.4) containing Hg^2+^ and Pb^2+^ with initial concentrations of 1, 2, 3, 4, 5, 6, and 8 mg·L^−1^ were added with 1 mg of MSN-SH and agitated in a rotator oscillator at 37 °C for 3 h to reach equilibrium. Then the solutions were sampled and analyzed as described above.

The adsorption capacity (Qe, mg·g^−1^), the amount of Hg^2+^ and Pb^2+^ adsorbed at equilibrium, was calculated according to the following equation.

(1) $$Q_{e}=\frac{V\left(C_{0}-C_{e}\right)}{m}\;$$

where C_0_ is the initial heavy metal concentration in the solution (mg·L^−1^), Ce is the equilibrium concentration (mg·L^−1^) after adsorption, V is the solution volume (L) and m is the mass of MSN-SH adsorbent (g).

The Langmuir equation (Langmuir, 1916) given in Equation (2) was applied to the data to provide an insight into the mechanism of the adsorption.

(2) $$\frac{C_{e}}{Q_{e}}=\frac{1}{Q_{0}b}+\frac{C_{e}}{Q_{0}}$$

where Q_0_ and b are Langmuir constants related to the maximum adsorption capacity and energy of adsorption, respectively.

### 3.7. Hemolysis Assays

The hemolysis assays were performed, according to a previous report [37]. Typically, the whole blood was diluted to 1/10 of its original volume using calcium and magnesium-free Dulbecco’s phosphate buffered saline (DPBS) solution. Red blood cells (RBCs) were isolated by centrifugation at 269 g for 10 min, washed, and re-suspended five times with PBS buffer solution (pH 7.4). Then, 0.2 mL of diluted RBCs suspension was added to 0.8 mL of PBS buffer solution containing different concentrations of MSNs-SS-DMSA and mixed by vortexing. RBCs samples treated with deionized water and DPBS were used as the positive and the negative control, respectively.

All the samples were kept in static conditions at room temperature for 3 h. Lastly, the mixtures were centrifuged at 6740 g for 3 min and their images were collected by a digital camera (IXUS 175, canon, Tokyo, Japan).

### 3.8. Cytotoxicity Assays

*In vitro* cytotoxicity of MSNs-SS-DMSA against HL-7702 cells was investigated by MTT assays. In addition, 2.0 × 10^4^ of HL-7702 cells were seeded on 96-well culture plates at 37 °C with 5% CO_2_. After 12 h of attachment, the culture medium was removed and the cells were mixed with mediums containing different concentrations of MSNs-SS-DMSA. After incubation for 24 h, mediums were removed and cells were treated with the MTT (2 mg·mL^−1^) solution. After the next 4 h of incubation, MTT-containing solution was removed and 150 mL DMSO was added to each well to dissolve formazan. The absorption was measured at a wavelength of 570 nm using a microplate reader (DNM−9602G, Perlong, city, if any state, country). The absorption due to the interaction between the nanoparticles and the MTT was removed in the data treatment.

### 3.9. Characterization

The morphologies and structures of the particle samples were investigated using a JEOL JSM−7401F field emission scanning electron microscope (SEM) and a JEM−2100F transmission electron microscopy (TEM). Zeta potential of the particle samples was measured using a Malvern Zetasizer Nano ZS90. The weight loss of the particle samples was determined by a TGA instrument (SDTQ 600, Shimadzu, Tokyo, Japan) at a scan rate of 10 °C·min^−1^ and in the temperature range of 20–800 °C. Nitrogen adsorption-desorption isotherms of the particle samples were measured at 77 K on a Micrometitics Tristar 3000 system. The chemical properties of the particle samples were characterized by Fourier transform infrared spectroscopy (FTIR) (Fivector, Bruker, Karlsruhe, Germay) and Raman spectroscopy (Renishaw inVia, London, England). Interactions between particles and GSH were monitored by a Fluorescence spectrometer (RF−6000, Shimadzu, Tokyo, Japan).

### 3.10. Statistical Analysis

Data were expressed as mean ± S.D. Statistical analysis was performed by the Student′s *t* test and one-way analysis of variance (ANOVA) followed by Dunnett′s *post hoc* test when appropriate. Differences were considered statistically significant at *p* < 0.05.

## 4. Conclusions

In this study, a novel nanocomposite composed of mesoporous silica nanoparticles. A disulfide bond and a dimercaptosuccinic acid were synthesized and characterized. The nanocomposite was found to be stable in extracellular environments and was internalized by cells. It released the dimercaptosuccinic acid via the cleavage of the disulfide bond and was compatible with blood and cells at a wider dosage range. Furthermore, its intracellular metabolite known as the thiolized mesoporous silica nanoparticles was verified to possess strong adsorbability for heavy metal models of Hg^2+^ and Pb^2+^, according to in vitro simulated tests. All information above could not only enable it to be a redox−responsive drug delivery system for delivery of dimercaptosuccinic acid but also to be a solution for defects and efficacy of this chelator. Thus, construction of this nanocomposite could present a new insight for chelation therapy. However, since the effect of a chelator involves many facets of assessments such as excretion or redistribution of heavy metals as well as extraction of essential elements [1], further investigations for detoxification performances of this nanocomposite using animal models are necessary and this work is underway in our library.

## Abbreviations

DMSA	dimmercaptosuccinic acid
MSNs	mesoporous silica nanoparticles
GSH	glutathione
DDS	drug delivery system
SGF	synthetic gastric fluid
SIF	synthetic intestinal fluid
CTAB	cetyltrimethyl ammonium bromide
TEOS	tetraethoxysilane
Py-SS-Py	2, 2-dipyridyl disulfide
DOX	doxorubicin
EDC	1-ethyl-3-(3-dimethylaminopropyl) carbodiimide hydrochloride
NHS	*N*-hydroxy-succinimide
FITC	fluorescein isothiocyanate
MPTES	3-mercaptopropyltriethoxysilane
APTES	3-aminopropyltriethoxysilane
FBS	Fetal calf serum
DMEM	Dulbecco′s modified eagle medium
CAS	Chinese Academy of Sciences
LSCM	laser scanning confocal microscope
SEM	scanning electron microscope
TEM	transmission electron microscopy
FTIR	Fourier transform infrared spectroscopy
ANOVA	analysis of variance
